# Enhanced sonophotocatalytic degradation of phthalate acid ester using copper-chromium layered double hydroxides on carbon nanotubes and biochar

**DOI:** 10.1016/j.ultsonch.2025.107351

**Published:** 2025-04-11

**Authors:** Tannaz Sadeghi Rad, Alireza Khataee, Emine Sevval Yazici, Erhan Gengec, Mehmet Kobya, Yeojoon Yoon

**Affiliations:** a Department of Environmental Engineering, Faculty of Engineering, Gebze Technical University, Gebze 41400, Türkiye; b Department of Chemical Engineering, Istanbul Technical University, Istanbul 34469, Türkiye; c Research Laboratory of Advanced Water and Wastewater Treatment Processes, Department of Applied Chemistry, Faculty of Chemistry, University of Tabriz, Tabriz 51666−16471, Iran; d Department of Environmental Protection, University of Kocaeli, Kocaeli 41275, Türkiye; e Department of Environmental Engineering, Kyrgyz-Turkish Manas University, Bishkek 720038, Kyrgyzstan; f Department of Environmental and Energy Engineering, Yonsei University, Wonju, Republic of Korea

**Keywords:** Biochar, Carbon nanotube, Layered double hydroxide, Advanced oxidation processes, Plasticizer

## Abstract

Layered double hydroxides (LDHs) are lamellar and stable nanocatalysts driven by visible light. They have received much attention in the context of advanced oxidation processes. Their catalytic performance is remarkably restricted owing to undesired aggregation and the possibility of electron-hole recombination. To address these issues, we engineered carbon-nanotube (CNT)-and biochar (BC)-based CuCr LDH nanocomposites via a facile hydrothermal method. The synthesized nanocomposites were physically and chemically characterized using various methods. The performances of the BC-CuCr LDH and CNT-CuCr LDH nanocomposites were compared during the sonophotocatalytic degradation of dimethyl phthalate. With 1.5 g L^-1^ of BC-CuCr LDH, complete degradation of dimethyl phthalate was achieved within 25 min under 50 W light intensity and 150 W ultrasound irradiation with a synergy factor of 14. The critical roles of the hydroxyl and superoxide radicals were confirmed by the addition of several inhibitors. Ultimately, six possible intermediates generated during the sonophotocatalytic process were identified using gas chromatography-mass spectrometry (GCMS).

## 1 Introduction

Dimethyl phthalate (DMP) is a phthalate acid ester and has been used extensively as a plasticizer to boost the elasticity, transparency, flexibility, and plasticity of plastics [1,2]. In addition, DMP is widely used in food packaging, personal care products, electronic equipment, and medical tools [3]. Owing to the weak chemical bonds between polymer substrates, hazardous DMP residues are gradually released into aquatic media, including oceans, rivers, groundwater, and drinking water, where they pose a threat to human health through metabolic inhibition, endocrine disruption, and oxidative damage [2,4]. Moreover, DMP is strongly hydrophobic and is easily adsorbed onto suspended particulates or phytoplankton, which then precipitate and accumulate in sediments [5]. Considering the persistent, detrimental, and bioaccumulative characteristics of DMP, increasing attention has been paid to its removal from water sources.

Biological treatment [6], adsorption [7], ozonation [8], UV irradiation [9], and flocculation [10] have all been suggested as methods for removing phthalates. However, the robust chemical structure of DMP prevents its effective disintegration using these methods [11]. Furthermore, numerous reports have shown that DMP cannot be degraded or mineralized using traditional physical methods [[12], [13], [14]]. Therefore, the development of an environmentally friendly and feasible technique for degrading DMP in water and wastewater sources is receiving much attention. Photocatalytic-based advanced oxidation processes (AOPs) have been identified as a suitable technology for removing refractory contaminants through the generation of reactive oxygen species (ROSs), as they not only eliminate contaminants but also mineralize them into CO_2_ and H_2_O [[15], [16], [17]]. Sonocatalysis is an effective AOP in which the generated cavitation bubbles, along with the heterogeneous catalyst, lead to the formation of ROSs [18,19]. Acoustic cavitation can cause shock waves and microstreaming, thus accelerating the chemical reactions [20]. The extreme temperature created by cavitation triggers the thermal dissociation of H_2_O, leading to the production of ROSs [21]. Hence, integrating sonication with photocatalysis (sonophotocatalysis) has emerged as a versatile approach to overcoming these limitations and alleviating the environmental crisis [22,23]. The synergistic advantages of this hybrid approach over separate methods have been explained in several reports [19,23].

Layered double hydroxides (LDHs) are a widespread class of nanolayered materials with a regular structure containing interlaminar positive and negative charges, conferring their ability as heterogeneous nanocatalysts which can be used in the sustainable treatment of propellant wastewater [24,25]. LDHs have received extensive attention because of their rheological characteristics, two-dimensional lamellar nanostructure, diverse chemical compositions, and ion-exchange properties [26]. Accordingly, LDHs can be applied in water treatment processes, including photocatalysis, Fenton-based processes, sonocatalysis, adsorption, and catalytic ozonation [25,27]. In particular, Cu-based LDHs have widespread applications owing to their intrinsic structure and favorable catalytic activity [28,29]. Nevertheless, the difficult separation of LDHs from solution and their undesired aggregation may restrict their potential application and induce secondary pollution issues [30]. Hence, LDHs should be anchored onto carbon-based materials such as biochar (BC) and carbon nanotubes (CNT) to promote their catalytic performance. These heterostructures can cause charge carrier mobility and promote electron-hole separation [31].

Therefore, we fabricated CuCr LDH material through the co-precipitation route and loaded it onto BC and CNT using the hydrothermal method. The structural characteristics of the synthesized catalysts were examined using X-ray diffraction (XRD), scanning electron microscopy (SEM), transmission electron microscopy (TEM), X-ray photoelectron spectroscopy (XPS), differential reflectance spectroscopy (DRS), Fourier transform infrared spectrometry (FTIR), and adsorption–desorption isotherms. The sonophotocatalytic activities of the nanocomposites were compared with those of DMP. To the best of our knowledge, such application of BC-CuCr LDH and CNT-CuCr LDH for the sonophotocatalytic degradation of DMP has not yet been reported. Besides, the synthesis and characterization of the BC-CuCr LDH and CNT-CuCr LDH were comprehensively evaluated for the first time.

## 2 Material and methods

Descriptions of the chemicals and characterization methods are provided in the Supplementary Data (**Text S1**).

### 2.1 Preparation of the nanocomposites

CuCr LDH was prepared using the method described in our previous study [29]. To prepare the BC-CuCr LDH and CNT-CuCr LDH nanocomposites, BC or CNT (0.08 g) was ultrasonically dispersed in distilled water. A solution containing 4.57  g of Cr(NO_3_)_3_·9H_2_O and 4.83  g of Cu(NO_3_)_2_·3H_2_O was provided under the nitrogen gas and the pH was set to 9 by the dropwise addition of NaOH. The dispersed carbonaceous solution was added to the basic solution and stirred. The mixture was kept at 90 °C for 24 h in an autoclave. The obtained suspension was rinsed with distilled water and dried at 60 °C.

### 2.2 Sonophotocatalytic tests

The provided nanocomposites were placed in an ultrasonic bath along with a DMP solution (Elmasonic P, Germany) and simultaneously subjected to Visible light (positioned 10 cm from the solution). A UV–Vis spectrophotometer (SU-6100, Perkin Elmer, USA) was used to measure the absorbance of samples taken from the solution at specific time intervals. Furthermore, the sonophotocatalytic degradation of DMP in a real wastewater matrix containing plasticizers in the presence of BC-CuCr LDH was explored. The obtained wastewater had the characteristics of pH ∼ 8, conductivity = 1903 μs cm^−1^, and.

TOC = 17 mg L^-1^. The electrocoagulation process was applied as a pretreatment step with aluminum electrodes, and a current of 1 A for 60 min.

## 3 Results and discussion

### 3.1 Characterizations of the synthesized nanocomposites

**Fig. 1:**
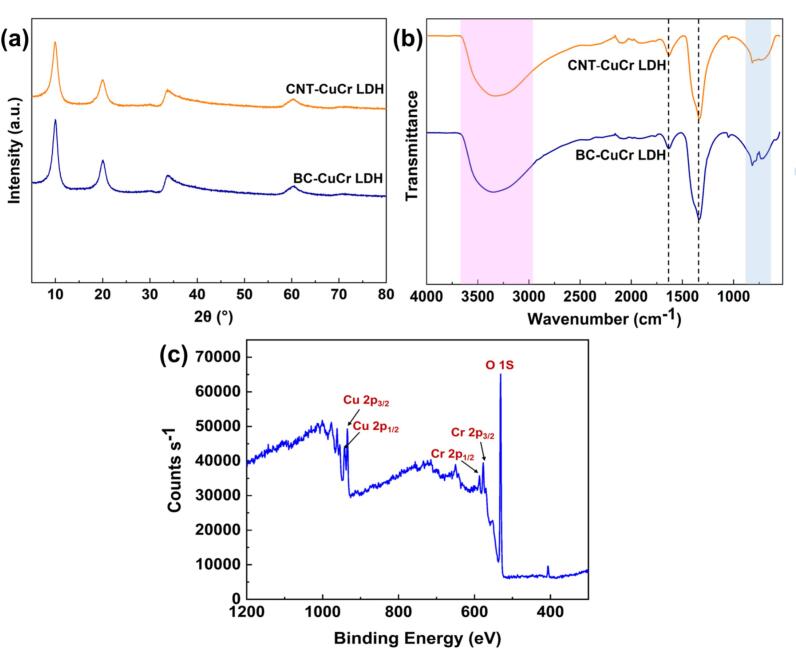
(a) XRD, (b) FTIR, and (c) wide XPS spectra obtained for CuCr LDH.

Fig. 1**a** shows the XRD patterns obtained for the BC-CuCr LDH and CNT-CuCr LDH nanocomposites. The XRD spectrum of bare CuCr LDH was reported in our previous paper [29]. In both XRD spectra, a peak appeared at a 2θ value of 10°, confirming the layered hexagonal structure as well as R − 3 m rhombohedral symmetry (JCPDS 00–035–0965) [32,33]. Patil et al. [33] reported similar peaks in XRD patterns obtained for CuCr LDH. There were no obvious peaks related to carbonaceous materials, owing to the uniform distribution of these phases without any agglomeration or phase separation [34]. No further phases were identified, confirming the high purity and effective synthesis of the BC-CuCr LDH and CNT-CuCr LDH samples. The broad humps at 2θ = 60° and 2θ = 33° reveal an in-plane hexagonal turbostratic crystal structure [33].

The main functional groups of the synthesized nanocomposites were examined using FTIR in the range of 400–4000  cm^−1^ (Fig. 1**b)**. Both the BC-CuCr LDH and CNT-CuCr LDH nanocomposites exhibited identical IR spectral patterns. The presence of Cu-O-Cu lattice vibrations was confirmed by the low-frequency spectral features below 1000  cm^−1^. The broad peaks at 3400 and 1670 cm^−1^ were assigned to the stretching vibration of the hydroxyl and the bending vibration of the H_2_O molecules, respectively [35]. Antisymmetric stretching of nitrate molecules is indicated by the sharp and strong peak at 1350 cm^−1^. FTIR analysis verified the presence of nitrate interlayer anions in the BC-CuCr LDH and CNT-CuCr LDH structures. Ziegenheim et al. [36] observed a similar trend in the FTIR spectra obtained for CuCr LDH.

XPS analysis was performed to further investigate the surface chemical composition of the CuCr LDH. Fig. 1**c** shows a full XPS spectrum of the pristine CuCr LDH sample. Two remarkable peaks were observed at approximately 954 and 935 eV, related to Cu 2p_1/2_ and Cu 2p_3/2_, respectively, indicating the divalent state of Cu. The peaks located at approximately 577 and 587 eV originate from Cr 2p_3/2_ and Cr 2p_1/2_. This implies that Cr exists in the form of Cr^3+^. Moreover, the deconvoluted peaks of the elements are mentioned in detail in our previous paper [29]. These XPS findings confirm the efficacious preparation of the CuCr LDH.

**Fig. 2:**
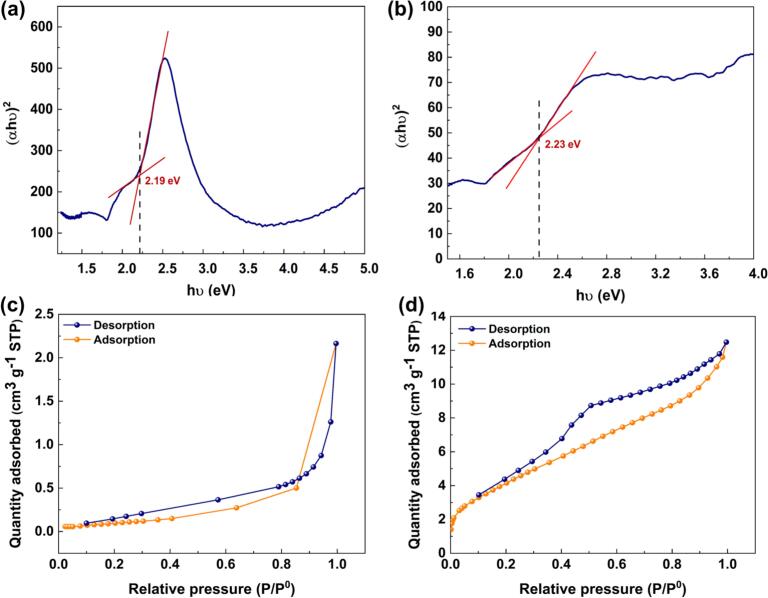
(αhν)^2^–hν curves (a and b) and N_2_ adsorption–desorption curves (c and d) for BC-CuCr LDH and CNT-CuCr LDH, respectively.

To examine the optical characteristics of the BC-CuCr LDH and CNT-CuCr LDH, (αhʋ)^2^-hʋ curves were obtained and are presented in Fig. 2**a and b**. The Tauc equation was used to compute the bandgap of the synthesized nanocomposites [37]. The band gaps of BC-CuCr LDH and CNT-CuCr LDH were calculated as 2.19 eV and 2.23 eV, respectively. These results reveal that the BC and CNT-based CuCr LDH nanocomposites can be sufficiently triggered by low-energy visible light and ultrasonic irradiation to form electron-hole pairs.

The nitrogen adsorption–desorption isotherms of the BC-CuCr and CNT-CuCr LDH nanocomposites were analyzed to determine their pore structures. As shown in Fig. 2**c**, the BC-CuCr LDH nanocomposite exhibits a type Ⅲ isotherm, indicating multilayer adsorption. The CNT-CuCr LDH exhibits a type IV isotherm, confirming the presence of slit-formed mesopores (Fig. 2**d)**. According to the BET data (Table. 1), the specific surface area for BC-CuCr LDH and CNT-CuCr LDH was computed as 0.5 and 13.5 m^2^ g^−1^, respectively. The lower amount of the specific surface area of the BC-containing sample may be attributed to the incorporation of restacked CuCr LDH nanosheets on the BC’s surface. Additionally, both nanocomposites have adsorption average pore diameter in the range of 2–50 nm, confirming the mesoporous structure of the samples. Also, BJH plot data was indicated in Table. 1, affirming BET’s findings. Similar trends have been reported by other researchers [38,39].

**Table. 1:** Surface characteristics of the nanocomposites, based on the BET and BJH plots.

Plots	Variables	BC-CuCr LDH	CNT-CuCr LDH
BET	Q_m_ [cm^3^(STP) g^−1^]	0.097	3.095
	a_s_, BET [m^2^ g^−1^]	0.5	13.5
	Adsorption average pore diameter [nm]	23.57	4.93
BJH	BJH adsorption cumulative volume of pores [cm^3^ g^−1^]	0.0007	0.0188
	BJH adsorption cumulative surface area of pores [m^2^ g^−1^]	0.63	17.38
	BJH adsorption average pore width [nm]	4.90	4.33

**Fig. 3:**
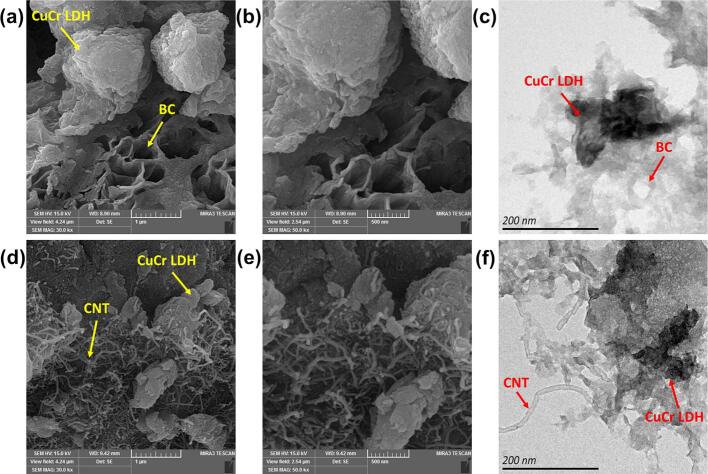
SEM images of (a and b) BC-CuCr LDH and (d and e) CNT-CuCr LDH and TEM images of (c) BC-CuCr LDH and (f) CNT-CuCr LDH.

The morphological characteristics of the CuCr LDH-based carbonaceous nanocomposites were examined using SEM and TEM (Fig. 3**a-f**). An SEM image of the BC-CuCr LDH sample reveals thin restacked CuCr LDH nanosheets along with a porous BC structure. In addition, these nanosheet structures were hybridized with nanotubes to form CNT-CuCr LDH nanocomposites. The TEM images show identical morphologies and structures for the BC-CuCr LDH and CNT-CuCr LDH nanocomposites, verifying the SEM findings.

### 3.2 Effect of disparate catalytic processes on degradation efficiency (DE%)

**Fig. 4:**
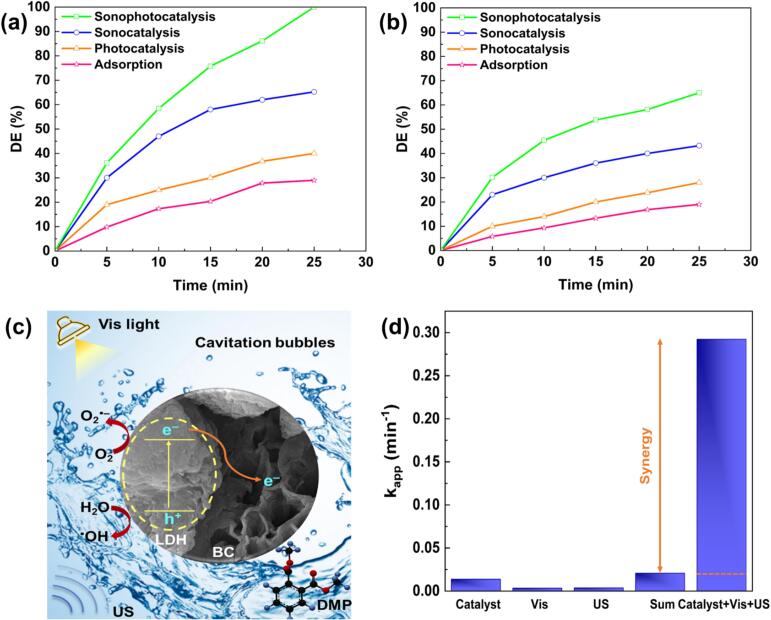
DE% values obtained for the degradation of DMP through sonophotocatalysis, sonocatalysis, photocatalysis, and adsorption by (a) BC-CuCr LDH and (b) CNT-CuCr LDH ([DMP]_0_ = 15 mg L^−1^, [BC-CuCr LDH] = 1.5 g L^−1^, pH = 8, light intensity = 50 W, and ultrasonic power = 150 W), (c) the sonophotocatalytic mechanism in the presence of BC-CuCr LDH, and (d) the synergistic impact obtained with BC-CuCr LDH.

To confirm the synergistic effect of the sonophotocatalytic process on DMP degradation, adsorption, photocatalysis, sonocatalysis, and sonophotocatalysis processes were performed in the presence of the synthesized samples. The DE% of the treatment processes in the absence of nanocomposites including sonophotolysis, photolysis, and sonolysis were comprehensively investigated in our previously published study [29]. Owing to the refractory nature of DMP, the aforementioned processes could not fully decompose the structure of this material. Therefore, heterogeneous catalysts play a decisive role in degrading the most robust compounds. Accordingly, the performance of adsorption, sonocatalysis, photocatalysis, and sonophotocatalysis in the presence of the synthesized nanocomposites was explored, as depicted in Fig. 4**a and b**. The adsorption of the contaminant molecules on BC-CuCr LDH and CNT-CuCr LDH resulted in the lowest DE%, revealing the limited capacity of the adsorption process to remove the contaminant. As shown in Fig. 4, photocatalysis in the presence of BC-CuCr LDH resulted in a higher DE% (41 %) than in the presence of CNT-CuCr LDH (29 %), which can be attributed to the lower bandgap energy (2.19  eV), as confirmed by DRS analysis. The functions of BC-CuCr LDH and CNT-CuCr LDH as sonocatalysts were also investigated. The sonocatalytic process proceeds via the generation and collapse of cavities, resulting in the formation of very high localized pressure and temperature. Therefore, cavitation produces highly oxidizing ROSs [40]. Along with turbulence, these ROSs can facilitate mass transfer and boost DE%. In addition, ultrasound waves can create electron-hole pairs in the presence of heterogeneous catalysts, enhancing the amount of ROSs. Ultimately, 25 min of sonocatalytic process in the presence of BC-CuCr LDH and CNT-CuCr LDH resulted in DE% values of 65.2 % and 43 %, respectively. Sonophotocatalysis, as a hybrid technique, combines semiconductor catalysts, light, and ultrasound irradiation, and could drastically reduce the time and energy required for DMP degradation [18]. Hence, the application of the BC-CuCr LDH and CNT-CuCr LDH nanocomposites as heterogeneous sonophotocatalysts was investigated. After 25 min of sonophotocatalytic process, DE% values of 100 % and 65 % were obtained in the presence of BC-CuCr LDH and CNT-CuCr LDH, respectively.

A possible mechanism for the sonophotocatalytic process is summarized in Fig. 4**c**. Generally, the BC-CuCr LDH nanocomposite demonstrated a higher DE% in all catalytic processes than the CNT-CuCr LDH nanocomposite. This can be attributed to the low bandgap and porous structure of the BC-based nanocomposite. The synergy factor of the process in the presence of the BC-based CuCr LDH is shown in Fig. 4**d** and was calculated using the equation reported in our previous study [29]. A synergy factor of 14 was achieved, which confirmed the remarkable interaction of light and ultrasonic irradiation with the heterogeneous catalyst in the deterioration of DMP. Additionally, **Table. S1** compares the obtained DE% values with those reported in similar studies. BC-CuCr LDH was selected as the sonophotocatalyst for further experiments.

### 3.3 Investigation of effective parameters

**Fig. 5:**
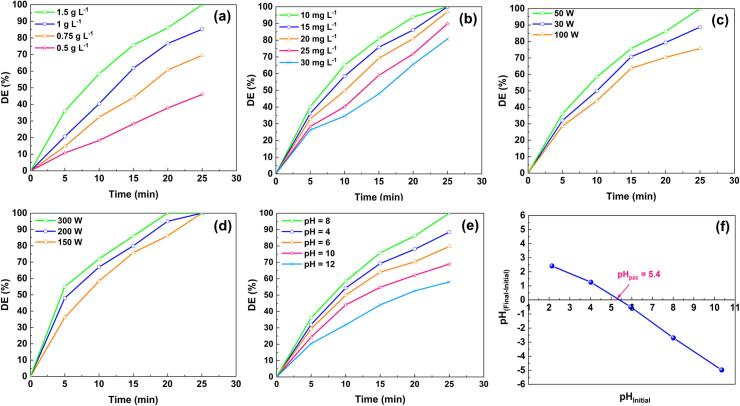
The influence of (a) BC-CuCr LDH dosage, (b) DMP concentration, (c) light intensity, (d) ultrasonic power, (e) pH, and (f) pH_pzc_ ([CuCr LDH/ BC] = 1.5 g L^−1^, [DMP]_0_ = 15 mg L^−1^, light intensity = 50 W, pH = 8 (natural pH of DMP), and ultrasonic power = 150 W) on the degradation of DMP.

The impact of the sonophotocatalyst concentration was investigated to determine the optimum dosage for achieving better DE%. A selection of data indicating the influence of different dosages of BC-CuCr LDH on DMP degradation is presented in Fig. 5**a**. As the sonophotocatalyst concentration increased from 0.5 to 1.5 g L^-1^, the DE% increased from 46 % to 100 %. This ascending trend can be attributed to an increase in the number of active sites on the catalyst, promoting the generation of high amounts of ROSs. In addition, numerous available sites on the catalyst surface would lead to more efficient absorption of light. Yun et al. [41] reported a similar upward trend in DE% with an increase in the catalyst dosage to 0.6 g L^-1^.

The DE% value obtained with the BC-based nanocomposite was investigated at different initial DMP concentrations, as shown in Fig. 5**b**. The removal efficiency of DMP declined from 100 % to 80.7 % as the DMP concentration was incremented. It should be noted that the available active sites of BC-CuCr LDH may become occupied by the generated intermediates or by high concentrations of DMP. These byproducts can compete with DMP molecules for ROS reactions, leading to a reduction in DE% [42]. Furthermore, ROS generation may be reduced by a decrease in penetrating light and ultrasound irradiations to the sonophotocatalyst surface.

Light intensity is a critical factor in photocatalytic-based processes, with an eminent impact on the formation of electron-hole pairs [43]. Fig. 5**c** shows the influence of the light intensity on the DE% of DMP. The efficacy of sonophotocatalytic decomposition of DMP increased from 88.7 % to 100 % when the light intensity was increased from 30 to 50 W. Remarkably, more photons were irradiated to activate the BC-CuCr LDH nanocomposite as the light intensity increased. However, as light intensity incremented after the saturation of photon flux, the enhancing impact of extreme photons is limited [44]. Hence, there was no improvement in the DE% at 100 W because the photon flux might be saturated. Also, Zhong et al. [44] reported a similar trend of the adverse effect of increasing the light intensity from 130 to 150 mw cm^−2^ on degradation profile.

A critical factor during the sonophotocatalytic decomposition of contaminants is the number of cavitation bubbles generated, which is mostly related to the ultrasonic power. With this aim, we examined the effect of ultrasonic power in the range 150 to 300 W on the removal of DMP through sonophotocatalysis, as depicted in Fig. 5**d**. Under all ultrasonic powers, the DE% reached 100 % within 25 min. As the ultrasonic power increased, the reaction time required to achieve complete decontamination decreased. As can be observed from Fig. 5**d**, the DE% reached 100 % within 20 min at an ultrasonic power of 300 W. The results revealed that high ultrasonic irradiation boosted cavitation bubbles, de-aggregation of the BC-CuCr LDH surface, and the formation of ROSs, resulting in a superb DE%. Based on economic concerns, 150 W was selected as the appropriate ultrasonic power for further runs.

pH is a notable effective parameter in AOPs which can transform the surface charge of the sonophotocatalyst or the state of the contaminant [41]. The efficacy of the sonophotocatalytic process in the presence of BC-CuCr LDH was examined at various initial pH values (4–12) (Fig. 5**e**). Complete degradation of DMP was achieved with an initial pH of 8. To account for these results, the pH_pzc_ of the BC-CuCr LDH nanocomposite was computed as 5.4 and is shown in Fig. 5**f**. At pH < pH_pzc_, the positive catalyst surface may attract the oxygenated or electron-enriched sites of DMP, confirming the beneficial impact of acidic conditions over basic conditions. DE% demonstrated a downward trend under basic conditions owing to the conversion of carbon dioxide molecules to carbonate ions. These carbonate ions may have consumed ^•^OH, leading to a decrease in DE%. Based on these results, we selected values for the ultrasonic power, catalyst dosage, pH, light intensity, and initial DMP concentration of 150 W, 1.5 g L^-1^, 8, 50 W, and 15 mg L^-1^, respectively, for subsequent tests.

### 3.4 Retrievability of BC-CuCr LDH

Constant exposure to photo- and/or ultrasonic irradiation tends to limit the performance of the nanocomposites. Thus, the reusability and durability of BC-CuCr LDH during the sonophotocatalytic process were evaluated in four consecutive tests. As shown in **Fig. S1a,** a decrease in DE% of approximately 14 % occurred after four cycles, which seems reasonable. In addition, the chemical nature of the reused BC-CuCr LDH was compared with that of pristine nanocomposites (**Fig. S1b**). The results revealed no major chemical alterations in the composition of BC-CuCr LDH. Therefore, BC-CuCr LDH is a persistent sonophotocatalyst, suitable for industrial applications.

### 3.5 Effect of scavengers

Aqueous media commonly contain diverse impurities, including organic salts, which can disrupt the reaction between contaminant molecules and the generated ROSs. Formic acid, p-benzoquinone (p-BQ), and isopropanol are scavengers that attenuate DE% by interacting with holes, superoxide radicals, and hydroxyl radicals, respectively. To investigate the impact of these reactive species on the DE% of DMP, various tests were performed in the absence and presence of scavengers, and the results are shown in **Fig. S1c**. An obvious decrease in DE% (approximately 32 %) was observed in the presence of p-BQ, revealing the major role of O_2_^•−^ radicals in DMP decomposition. In the presence of isopropanol, the DE% decreased, reaching only 83 % after 25 min, which can be attributed to the contribution of ^•^OH radicals. In addition, the photo-generated holes were captured by formic acid molecules, leading to a reduction in DE%. The impact of reactive species during sonophotocatalysis is denoted as O_2_^•−^ > ^•^OH > holes. These results agree with those of Mishra et al. [45].

### 3.6 Degradation of DMP in real wastewater and reaction intermediates

With the aim of comparing the obtained results with the real condition, sonophotocatalytic process in the presence of BC-CuCr LDH was examined in a real wastewater matrix. Also, various organic/inorganic structures can be detected in real wastewater that compete with DMP molecules to react with ROSs. Hence, the performance of BC-CuCr LDH nanocomposite for the treatment of plasticizer-containing wastewater under the optimized condition was studied. As a pretreatment procedure, electrocoagulation process was applied. About 33.5 % of the mineralization was obtained after 60 min of electrocoagulation. During 360 min of sonophotocatalytic process in the presence of BC-CuCr LDH, 60.4 % of the real wastewater was mineralized. It has been discovered that BC-CuCr LDH is capable of mineralizing the plasticizer-containing wastewater.

**Fig. 6:**
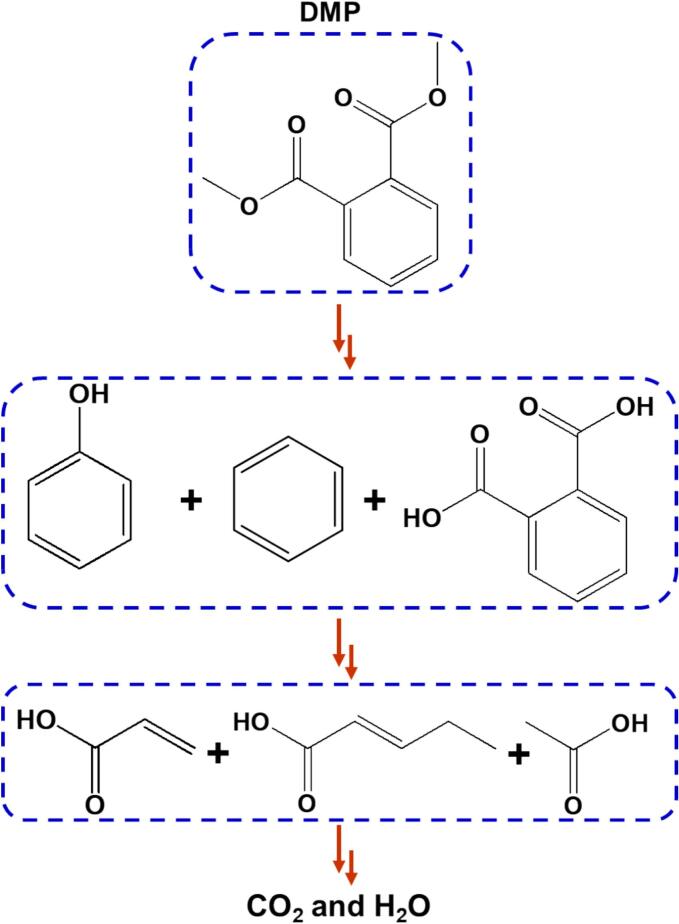
The plausible reaction pathway for the decomposition of DMP.

Gas chromatography-mass spectrometry (GCMS) analysis was performed to detect the byproducts generated during sonophotocatalysis. As depicted in Fig. 6 **and Table S2**, six main intermediates were formed during the reaction. First, the ester groups of DMP were hydrolyzed by the generated reactive species such as holes, hydroxyl groups, and superoxide radicals to generate aromatic organic compounds. Subsequently, several simple-structured carbocyclic acids were formed, which may have undergone further mineralization to produce CO_2_ and H_2_O molecules. Furthermore, some byproducts may not have been identified owing to rapid oxidation.

### 3.7 An overall mechanism for sonophotocatalytic decomposition of DMP

The sonophotocatalytic disintegration of DMP degradation in the presence of BC-CuCr LDH is substantially based on the synergistic interaction among BC and CuCr LDH owing to the formation of electron-holes, transferring electrons on the BC surface, and the high generation of ROSs. BC-CuCr LDH has the capability of adsorbing diverse organic compounds, due to the presence of the oxygenated functional groups on its active sites, which was well confirmed by FTIR analysis. The degradation process was initiated by the adsorption of DMP on the catalyst surface and continued by a reaction between adsorbed DMP molecules and the sono/photogenerated ROSs.

Under light irradiation, electrons in the valence band of CuCr LDH were migrated to the conduction band resulting in holes’ development in the valence band **(Eq.** (1). It is feasible to hinder the electron-hole’s recombination via transferring the excited electrons from the valence band of CuCr LDH to the pores and active surfaces of the BC **(Eq.** (2).

(1) $$\text{CuCr LDH}\;\overset{\text{light irradiation}}{\to }\;\text{CuCr LDH (}\text{holes}+\text{electrons}\text{)}$$

(2) CuCr LDH (holes+electrons) + BC→CuCr LDH (holes) + BC (electrons)

Along with light irradiation, ultrasonic waves can assist the decomposition of DMP through effective pathways including; 1) transferring the DMP molecules to the surface and pores of BC, (2) cleansing the nanocomposite surface from the degradation intermediates, (3) boosting the mass transfer of DMP among the bulk solution and the nanocomposite’s surface. Meanwhile, ultrasound waves are able to produce pressure bubbles and high temperatures. Besides, BC-CuCr LDH as a heterogeneous sonophotocatalyst can boost cavitation by providing extra nuclei for bubble formation. Improving cavitation can result in enhancing water pyrolysis and H^•^, ^•^OH, and HO_2_^•^ radical production (**Eqs.** (3), (4).

(3) H_2_O→OH^.^+H^.^

(4) O_2_+H^.^→HO_2_^.^

According to the findings obtained by the addition of scavengers, it can be concluded that superoxide radicals play a fundamental role in the decomposition of DMP molecules adsorbed onto the BC-CuCr LDH nanocomposite, which can be generated by the reaction among the adsorbed oxygen with the excited electrons **(Eq.** (5). Additionally, hydroxyl radicals and holes can involve in the degradation of DMP to form intermediates.

(5) O_2_+BC (electrons)→O_2_^⋅−^+BC

Eventually, it can be concluded that reactive radical species, holes, adsorption of DMP to the nanocomposite surface, and ultrasonic and light irradiations assist the mineralization of DMP to diverse simple-structured acids, which may lead to the generation of safer intermediates as confirmed by GCMS analysis.

## 4 Conclusion

In this study, BC-CuCr LDH and CNT-CuCr LDH nanocomposites were fabricated, comprehensively characterized, and tested for their efficacy in degrading DMP via sonophotocatalysis. Based on XRD and FTIR results, the successful formation of BC-CuCr LDH and CNT-CuCr LDH nanocomposites was confirmed. The favored application of CuCr LDH nanosheets inside and outside the pores of BC and on the CNTs was confirmed by SEM and TEM images. Within 25 min, complete degradation (100 %) of DMP molecules was achieved with a DMP concentration of 15 mg L^-1^ in the presence of BC-CuCr LDH (1.5 g L^-1^) under 150 W ultrasonic power along with 50 W light irradiation. Scavenger tests indicated that hydroxyl and superoxide radicals were the most prominent species in the sonophotocatalytic disintegration of DMP molecules. The factors influencing the sonophotocatalytic process were thoroughly assessed and optimized for further experiments. Additionally, the durability of the BC-CuCr LDH sonophotocatalyst was verified by four successive experiments, revealing a 14 % decrease in the DE%. A tentative DMP degradation pathway was proposed based on the GCMS outcomes. This study provides an alternative route for the synthesis of effective carbon-enriched nanocomposites for environmental remediation. The simultaneous decomposition of multiple refractory pollutants and thorough evaluation of the attenuation of power and intensity of ultrasound and photons in large-scale applications should be the focus of future research.

## CRediT authorship contribution statement

**Tannaz Sadeghi Rad:** Data curation, Formal analysis, Investigation, Writing – original draft. **Alireza Khataee:** Writing – review & editing. **Emine Sevval Yazici:** Formal analysis, Investigation, Writing – review & editing. **Erhan Gengec:** Data curation, Formal analysis, Investigation, Writing – original draft. **Mehmet Kobya:** Investigation, Writing – review & editing. **Yeojoon Yoon:** Investigation, Methodology, Writing – review & editing.

## Declaration of competing interest

The authors declare that they have no known competing financial interests or personal relationships that could have appeared to influence the work reported in this paper.
