# Supplementary material for: Enhanced sonophotocatalytic degradation of phthalate acid ester using copper-chromium layered double hydroxides on carbon nanotubes and biochar
Source: Ultrason Sonochem. 2025 Apr 11;117:107351. doi: 10.1016/j.ultsonch.2025.107351 (PMC12023866; doi:10.1016/j.ultsonch.2025.107351)
Supplement: Supplementary Data 1 [file mmc1.docx]

**Supplementary data**

**Enhanced sonophotocatalytic degradation of phthalate acid ester using copper-chromium layered double hydroxides on carbon nanotubes and biochar**

**Text S1. Characterizations**

All the reagents used in this study were sourced from Merck Co. (Germany) and applied without extra purification. A plasticizer-containing wastewater sample was provided by the Gebze Organized Industrial Zone in Turkey.

SEM (MIRA 3, Tescan, Czech Republic) and TEM (JEM-2100, Jeol, Japan) analyses were utilized to illustrate the morphology of the so-prepared catalysts. XPS and adsorption-desorption isotherms were recorded by Kratos AXIS UltraDLD (Japan) and Mini II, BELSORP (Japan), respectively. The functional groups and the crystal structures of the BC-CuCr LDH and CNT-CuCr LDH were determined by FTIR (Perkin Elmer Spectrum 100, Germany) and XRD (D8 Advance, Bruker, Germany) instruments, respectively. To determine the optical properties of the samples, UV-Vis DRS (Shimadzu UV-2600, Japan) was used. GCMS was analyzed by Agilent 6890 N (USA) to specify the generated by-products during the treatment process.

**
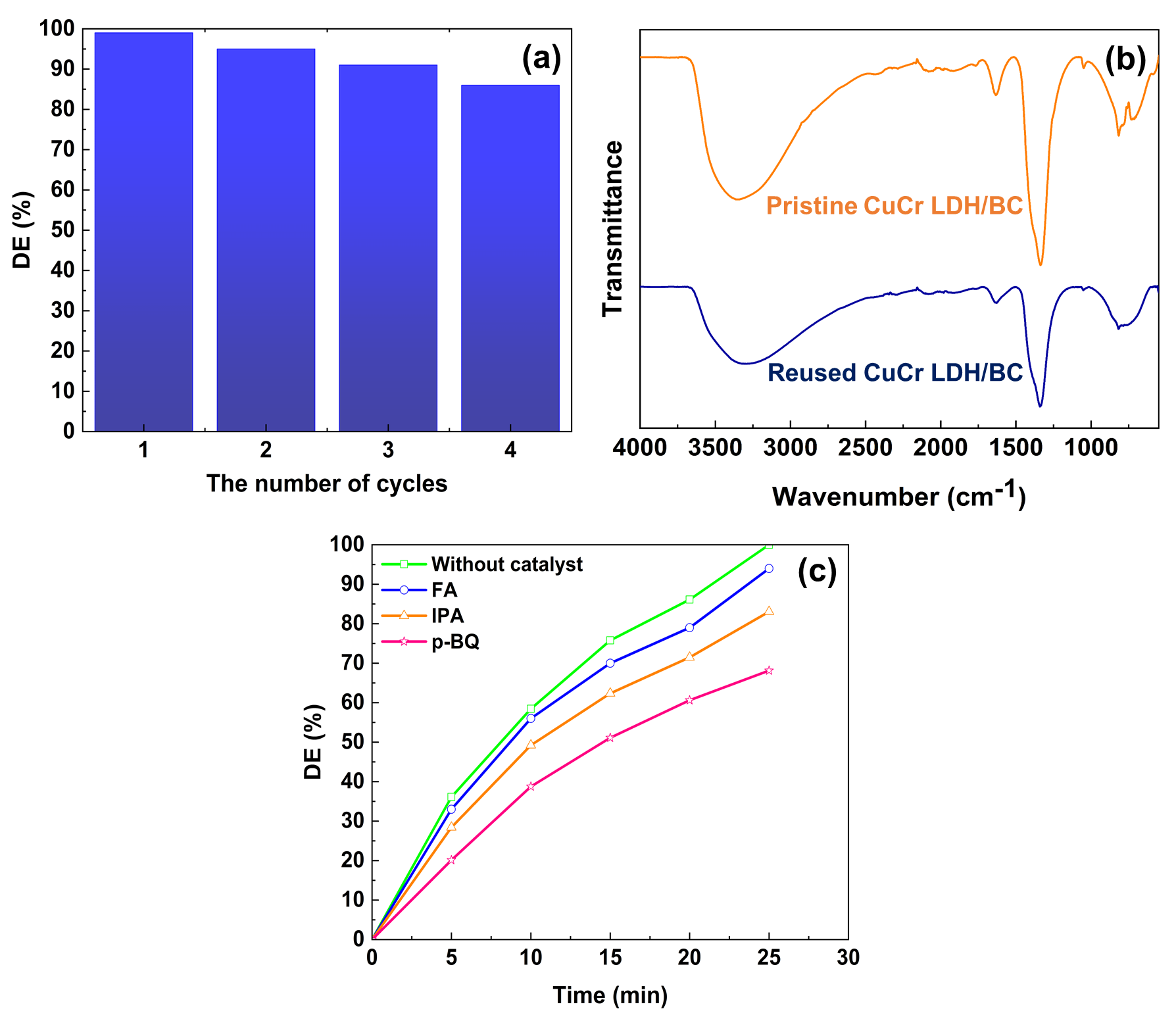
**

**Fig. S1.** (a) The retrievability of BC-CuCr LDH, (b) The FTIR spectra of the pristine and reused BC-CuCr LDH, and (c) The effect of different scavengers on the DE%.

**Table S1.** The sonophotocatalytic performance of diverse nanocomposites for the degradation of persistent contaminants.

| Catalysts | Reaction condition | DE% | | Refs. |
| --- | --- | --- | --- | --- |
| ZnO/carbon nanotube | [4-nitrophenol] = 10 mg L^−1^, [Catalyst] = 1.75 g L^−1^, ultrasonic power = 60 W, light intensity = 160 W , pH = 4, [H_2_O_2_] = 40 mmol L^-1^, and time = 180 min | 97% | [1] | |
| Sporopollenin/ZnO | [Dimethyl phthalate] = 10 mg L^-1^, [Catalyst] = 0.1 g L^−1^, ultrasonic power = 300 W, light intensity = 50 W, and time = 120 min | 62.5% | | [2] |
| ZnFe LDH/rGO/g-C_3_N_5_ | [Ciprofloxacin] = 10 mg L^-1^, [Catalyst] = 0.4 g L^−1^, ultrasonic power = 300 W, light intensity = 250 W, pH = 5.3, and time = 120 min | 95% | | [3] |
| Bi_2_MoO_6_/FeVO_4_ | [Ciprofloxacin] = 10 mg L^−1^, [Catalyst] = 0.5 g L^−1^, [H_2_O_2_] = 20 mmol L^-1^, light intensity = 36 W, ultrasonic power = 50 W, and time = 200 min | 93.43% | | [4] |
| BC-CuCr LDH | [Dimethyl phthalate] = 15 mg L^−1^, [Catalyst] = 1.5 g L^−1^, ultrasonic power = 150 W, light intensity = 50 W, pH = 8, and time = 25 min | 100% | | Our  study |

**T****able S2.** By-products formed during the degradation of DMP by sonophotocatalysis. ([DMP]_0_ = 15 mg L^−1^, [BC-CuCr LDH] = 1.5 g L^−1^, pH = 8, US power = 150 W, and light intensity = 50 W).

| No. | Structures | Retention time  (min) | Main fragments (m/z)/(percent) |
| --- | --- | --- | --- |
| 1 |  | 5.563 | 75.10 (100%), 116.10 (80.89%), 73.10 (14.38%), 117.10 (8.09%), 76.10 (7.69%) |
| 2 |  | 6.942 | 147.10 (100%), 73.10 (77.13%), 217.10 (23.26%), 188.10 (20.68%), 148.05 (15.69%) |
| 3 | **** | 9.139 | 142.10 (100%), 158.00 (90.82%), 75.10 (87.97%), 57.10 (46.63%), 99.10 (44.53%) |
| 4 | **** | 13.877 | 163.10 (100%), 77.10 (15.53%), 164.00 (10.69%), 76.10 (9.60%), 92.00 (8.15%) |
| 5 |  | 14.609 | 205.20 (100%), 57.10 (54.73%), 71.10 (39.38%), 70.10 (27.35%), 70.10 (27.35%), 55.10 (27.22%) |
| 6 |  | 23.364 | 239.10 (100%), 240.10 (24.38%), 73.00 (21.64%), 207.00 (14.11%), 372.10 (11.90%) |

**References**

[1] M. Khairy, E.M. Naguib, M.M. Mohamed, Enhancement of photocatalytic and sonophotocatalytic degradation of 4-nitrophenol by ZnO/graphene oxide and ZnO/carbon nanotube nanocomposites, J. Photochem. Photobiol. Chem. 396 (2020) 112507. https://doi.org/10.1016/j.jphotochem.2020.112507.

[2] P. Yekan Motlagh, B. Vahid, S. Akay, B. Kayan, Y. Yoon, A. Khataee, Ultrasonic-assisted photocatalytic degradation of various organic contaminants using ZnO supported on a natural polymer of sporopollenin, Ultrason. Sonochem. 98 (2023) 106486. https://doi.org/10.1016/j.ultsonch.2023.106486.

[3] Y. Yea, S.SD. Elanchezhiyan, R. Saravanakumar, G. Jagan, J.U. Choi, K. Saravanakumar, C.M. Park, All-solid-state Z-scheme ZnFe–LDH/rGO/g-C_3_N_5_ heterojunction for enhanced sonophotocatalytic degradation of ciprofloxacin: Performance and mechanistic insights, Environ. Res. 247 (2024) 118209. https://doi.org/10.1016/j.envres.2024.118209.

[4] G. Fan, X. Lin, S. Yang, B. Du, Y. Lu, X. Huang, J. Wu, K.-Q. Xu, Design of continuous flow membrane reactor for in-situ sonophotocatalytic degradation of ciprofloxacin, J. Environ. Chem. Eng. 10 (2022) 108888. https://doi.org/10.1016/j.jece.2022.108888.
